# Genome-Wide Comparative Analysis of Flowering-Time Genes; Insights on the Gene Family Expansion and Evolutionary Perspective

**DOI:** 10.3389/fpls.2021.702243

**Published:** 2021-07-05

**Authors:** Seongmin Hong, Yong Pyo Lim, Suk-Yoon Kwon, Ah-Young Shin, Yong-Min Kim

**Affiliations:** ^1^Genome Editing Research Center, Korea Research Institute of Bioscience and Biotechnology (KRIBB), Daejeon, South Korea; ^2^Molecular Genetics and Genomics Laboratory, Department of Horticulture, College of Agriculture and Life Sciences, Chungnam National University, Daejeon, South Korea; ^3^Plant Systems Engineering Research Center, Korea Research Institute of Bioscience and Biotechnology, Daejeon, South Korea

**Keywords:** flowering-time gene, whole genome duplication, copy number variation, polyploidization, functional diversification

## Abstract

In polyploids, whole genome duplication (WGD) played a significant role in genome expansion, evolution and diversification. Many gene families are expanded following polyploidization, with the duplicated genes functionally diversified by neofunctionalization or subfunctionalization. These mechanisms may support adaptation and have likely contributed plant survival during evolution. Flowering time is an important trait in plants, which affects critical features, such as crop yields. The flowering-time gene family is one of the largest expanded gene families in plants, with its members playing various roles in plant development. Here, we performed genome-wide identification and comparative analysis of flowering-time genes in three palnt families i.e., Malvaceae, Brassicaceae, and Solanaceae, which indicate these genes were expanded following the event/s of polyploidization. Duplicated genes have been retained during evolution, although genome reorganization occurred in their flanking regions. Further investigation of sequence conservation and similarity network analyses provide evidence for functional diversification of duplicated genes during evolution. These functionally diversified genes play important roles in plant development and provide advantages to plants for adaptation and survival in response to environmental changes encountered during evolution. Collectively, we show that flowering-time genes were expanded following polyploidization and retained as large gene family by providing advantages from functional diversification during evolution.

## Introduction

Flowering time is an important trait in plants that has wide-ranging effects on features such as growth, development, and crop yields (Huang et al., 2011), and evolution of new crop types (Gill et al., 2021). Thus, numerous studies using approaches that include genetic mapping, T-DNA insertional mutagenesis, and functional study have been conducted on flowering-time genes in *Arabidopsis thaliana*, as well as many other important crops, such as Chinese cabbage (Lagercrantz et al., 1996), soybean (Tasma and Shoemaker, 2003), and maize (Buckler et al., 2009), before large amounts of genome data were available. Recently, pan-genome analysis of *Brassica napus* also provides insights of flowering time of oilseed rape (Song J.M. et al., 2020). In addition, genome-wide association studies of flowering-time genes have been performed in *Brassica juncea* (Akhatar et al., 2021), *Brassica napus* (Xu et al., 2020) and *Brassica rapa* (Gao et al., 2017). Critically, although a significant amount of information on flowering-time genes has been uncovered in *A. thaliana*, identification of orthologous genes—a first step for studies on flowering-time genes in crop plants—has been hindered by the lack of genome information.

Since the development of next-generation sequencing (NGS) technologies in the mid-2000s, whole-genome sequencing has become standard practice in biology, yielding valuable insights in numerous fields of study, including medicine, agriculture, biotechnology, and basic-science research. The technological advances allowing whole-genome sequencing using long-read data have facilitated the accumulation of large amounts of genome information, with high fidelity (Kersey, 2019). This large-scale acquisition of genomic information has further enabled the identification of conserved genes of interests for comparative genomic studies in many different organisms. For example, the genome-wide identification of flowering-time genes by bioinformatic analysis was reported in *A. thaliana* (Bouche et al., 2016) and *Gossypium raimondii* (Grover et al., 2015). Similarly, genome-wide identification and comparative analysis of cold-related genes (CRG) was performed in Brassicaceae and Malvaceae family members, which contain polyploid genomes (Song X.M. et al., 2020). This study uncovered a total of 420 CRG analogs of 115 *A. thaliana* CRGs *via* BlastP search and provided evidence that recursive polyploidization contributes to copy number expansion of CRGs (Song X.M. et al., 2020). Notably, flowering-time genes were also found to be expanded and conserved in high copy numbers by recursive polyploidization with subsequent diploidization in *Hibiscus syriacus*, a member of the Malvaceae family (Kim Y.M. et al., 2017).

Whole-genome duplication (WGD), also known as polyploidization, is an important mechanism for speciation (Soltis et al., 2015), with approximately 15% of speciation events involving this process (Wood et al., 2009). WGD also represents one of the major mechanisms for gene duplication, along with processes such as segmental duplication, tandem duplication and retroduplication. In particular, many polyploid genomes have been reported in the Malvaceae family, including *G. hirsutum* (A_*t*_D_*t*_), *G. barbadense* (A_*t*_D_*t*_), *G. mustelinum* (A_*t*_D_*t*_), *G. tomentosum* (A_*t*_D_*t*_), *G. darwinii* (A_*t*_D_*t*_), *Durio zibethinus*, and *H. syriacus*. Thus, genomes belonging to Malvaceae family members are a valuable resource for investigating the relationship between WGD and copy number variation for flowering genes.

Here, to further explore this relationship, we performed a comprehensive comparative analysis of flowering-time genes in 19 plant species within the Malvaceae, Brassicaceae, and Solanaceae families. In total, 22,784 flowering-time genes were identified by their domain architectures. We found that genes involved in flowering time are highly conserved, and most of these genes were duplicated following WGD. In some cases comparison of diploid and polyploid genomes revealed that flowering-time genes are conserved, although structural genomic modifications that occurred after polyploidization, such as the active proliferation of repetitive sequences or gene insertions (Song J.M. et al., 2020), were detected in their flanking regions. Similarity network analysis further suggests that flowering-time genes have been functionally diversified during the course of evolution.

## Materials and Methods

### Collection of Plant Genomic Data

Genomic data from *T. cacao* (Argout et al., 2011), *G. raimondii* (Wang K. et al., 2012), and five allopolyploid *Gossypium* species (Chen et al., 2020) were downloaded from Phytozome v.12. Genomic data from *G. arboreum* (Li et al., 2014) and *Gossypium turneri* (Udall et al., 2019) were downloaded from CottonGen. Genomic data from *Corchorus capsularis* (Islam et al., 2017) are available in Ensembl plant. Proteins sequences from *Herrania umbratica^1^* and *D. zibethinus* (Teh et al., 2017) were downloaded from the National Center for Biotechnology Information (NCBI). Genomic data from the three *Brassica* species included in these analyses (Chalhoub et al., 2014; Liu et al., 2014; Zhang et al., 2018) were downloaded from the *Brassica* database (BRAD). For *A. thaliana*, ARAPORT 11 annotation data were downloaded from The *Arabidopsis* Information Resource (TAIR) database (Cheng et al., 2017). Genomic data from *Solanum lycopersicum* (Tomato Genome, 2012) and *Capsicum annuum* (Kim et al., 2014) were downloaded from the Sol Genomics Network and Pepper Genome Database, respectively. *H. syriacus* (Gangneung) genomic data were released in 2017 (Kim Y.M. et al., 2017).

### Preparation of Protein Sequences

Proteins containing asterisks within their sequences, other than those at the end of each protein, were first removed by an in-house script. Redundant protein sequences were then filtered for each species by CD-HIT (Li and Godzik, 2006). To remove alternative splicing variant sequences, primary transcript sequences were retrieved from the Phytozome database. In addition, two NCBI-derived protein sequences were filtered by confirming gene2accessions files.

### Identification of Flowering-Time Genes in Malvaceae, Brassicaceae, and Solanaceae

To identify flowering-time genes in plants belonging to the Malvaceae, Brassicaceae, and Solanaceae families, BlastP searches (E-value < 1 × 10^–10^ and amino acid identity > 60%) were performed using *A. thaliana* flowering-time genes in the FLOR-ID database (Bouche et al., 2016) as queries. To apply the previously published domain architecture-based method for identifying flowering-time genes (Koo et al., 2020), domain architectures of *A. thaliana* flowering-time genes in the FLOR-ID database were determined using InterPro (Mitchell et al., 2019) (interproscan.sh -i input_fasta_file -goterm -pa -f tsv -b result_file_name). These domain architectures were then used to identify flowering-time genes in Malvaceae, Brassicaceae, and Solanaceae as reported in previous study (Koo et al., 2020). Lastly, candidate genes not satisfying pre-established criteria (amino acid lengths > 90% shorter than the query gene or > 110% longer than the query gene) were filtered out, as described previously (Koo et al., 2020).

### Calculation of Conservation Scores

Multiple sequence alignment (MSA) was performed to calculate the best match for each flowering-time gene type having at least three copies in the 19 test species, using ClustalW (Thompson et al., 1994). The following parameters were used for MSA analysis: scoring matrix = BLOSUM 62, opening gap penalty = 10, end gap penalty = 10, extending gap penalty = 0.05. The conservation score was calculated with an in-house script for each flowering-time gene type.

### Detection of Whole Genome Duplication Patterns for Flowering-Time Genes

Duplication pattern identification analysis for flowering-time genes was conducted using gene datasets from chromosome-level assemblies. In the first step, MCscanX (Wang Y. et al., 2012) analysis was performed with all versus all BlastP results (E-value < 1.0 × 10^–5^, max target sequences = 2). The output scores were then used to classify each gene as single, dispersed, proximal, tandem, or WGD. In addition, sequence collinearity on the subgenome between diploid and polyploid genomes was calculated for *FCA* and *VIP5*. We performed calculations for identified collinear blocks to determine the following properties: total number of genes, genes without collinearity, and intergenic region proportion. LTRs in collinear block were identified by LtrDetector (Valencia and Girgis, 2019).

### Microsynteny of *Gossypium* Genomes

Proteins identified as having one copy in the AD genome diploid ancestor (*G. arboreum* and *G. raimondii*) and two copies in polyploid genomes (five species) were selected for microsynteny analysis. Genome regions containing *FCA* and *VIP5* along with their 20 adjacent genes were extracted using gff3 files for each species. Syntenic regions in the five allopolyploid *Gossypium* genomes were then identified by pblat (Wang and Kong, 2019). LTRs within syntenic regions were evaluated in all seven species by LtrDetector (Valencia and Girgis, 2019). Repeat density within syntenic regions was calculated using released repeat gff3 format files. Protein similarities between predicted genes within syntenic regions were calculated by pairwise BlastP analysis. Pairs fulfilling the following conditions were classified as conserved: E-value < 1 × 10^–10^, amino acid identity > 60%, coverage > 60%. Calculated properties of *Gossypium* synteny were visualized by Circos (Krzywinski et al., 2009) and RIdeogram (Hao et al., 2020).

### Sequence Similarity Network and Phylogenetic Tree Analyses

Sequence similarity networks for all FCA and VIP5 protein sequences identified by their domain architecture were constructed using the EGN pipeline, based on reciprocal BlastP analysis, using default parameters (Halary et al., 2013). Constructed networks were visualized by Cytoscape v.3.8.2 (Shannon et al., 2003). The same protein sequence dataset was used for phylogenetic tree analysis with MEGA X (Kumar et al., 2018). MSAs were generated with Muscle, and minimum evolution (ME) trees were constructed using the Jones-Taylor-Thornton (JTT) model and 500 bootstrap samples.

#### Estimation of Synonymous Nucleotide Substitutions at Synonymous Sites (Ks)

Individual flowering-time genes in each species were clustered using OrthoFinder (Emms and Kelly, 2015) and all-by-all alignments of the coding sequences were carried out using CLUSTAL OMEGA program (Sievers and Higgins, 2018). Protein alignments were subsequently translated to codon alignments based on their CDS using PAL2NAL (Suyama et al., 2006). Then, Ks value were estimated using codeml with F3 × 4 model in PAML 4 package (Yang, 2007).

## Results

### Identification of Flowering-Time Genes in the Malvaceae, Brassicaceae, and Solanaceae Families

To identify flowering-time genes in plants belonging to the Malvaceae, Brassicaceae, and Solanaceae families, we performed BlastP analysis using *A. thaliana* genes in the FLOR-ID database (Bouche et al., 2016) as queries (Table 1). In a previous study, CRGs were identified *via* a similar BlastP strategy (e.g., E-value cutoff < 1 × 10^–10^ and amino acid identity cutoff > 60%) in plants belonged to Brassicaceae and other families, including Malvaceae and Poaceae (Song X.M. et al., 2020). Here, we applied this method to identify flowering-time genes in 13 species of Malvaceae, four species of Brassicaceae, and two species of Solanaceae (Supplementary Tables 1–3). However, 147 of 295 *A. thaliana* genes could not be identified with this BlastP approach in Malvaceae, whereas most (279 of 295 genes) were identified in other Brassicaceae species (Supplementary Table 1). In a previous study, Brassicaceae and Malvaceae were estimated to have diverged approximately 91.91 Mya (Kim Y.M. et al., 2017). Thus, increased sequence variation in Malvaceae flowering-time gene homologs resulting from this large evolutionary distance between *A. thaliana* and Malvaceae species might be the reason they were unable to be detected by BlastP analysis.

**TABLE 1:** Classification of flowering genes by their functions in the FLOR-ID database.

**Category**	**Subcategory**	**Count**	**Gene symbols**
Aging		4	RAP2.7, SPL9, TOE2, and TPL
Ambient temperature		2	TPL, FCA
Circadian Clock		5	ELF4, LHY, PRR7, SRR1, and ZTL
General process and autonomous pathway	Biotic stress	1	PUB13
	Cell cycle and DNA replication	2	GIS5, TIL1
	Chromatin modifications	37	AHL22, ASHH1, ATX1, ATX2, BRM, CHC1, CLF, EFS, EMF2, FCA, FLD, FVE, FWA, HAM1, HDA05, HMG, HTA11, HUB2, INO80, JMJ14, JMJD5, LDL2, MBD9, MOS1, MRG1, MRG2, MSI1, PIE1, REF6, SDG25, SEF, SUVR5, TFL2, TRO, VEL1, VIM1, and VIM2
	Control of transcription	7	CCT, CDKC2, GCT, NDX, SPT16, VIP3, and VIP5
	mRNA & microRNA processing	18	ABH1, CBP20, CSTF64, CSTF77, DCL1, DCL3, DCL4, FLK, FPA, FY, GRP2, HLP1, HUA2, HULK2, LIF2, RRP6L1, RRP6L2, and SUS2
	Multiple processes	1	RCD1
	Protein stability control	7	CUL4, J3, SIZ1, UBC1, UBP12, UBP26, and ULP1D
	Unknown processes	1	LD
Hormones		5	ATH1, GA1, GA20OX3, GID1B, and RGA1
Photoperiodism, light perception, and signaling		34	AFR2, AGL15, AGL18, AS1, AT-STUbl4, BBX19, CDF2, CIB2, CO, COP1, CPK6, CRY1, CRY2, EBS, ELF4, FAR1, FD, FTIP1, HB16, HOS1, LHY, MYR1, NF-YC4, PHYB, PHYC, PHYD, RAP2.7, SPA1, SRR1, STO, TEM1, TOE2, TPL, and ZTL
Sugar		8	ADG1, HXK1, KIN10, NUC, PGM, SUC9, SUS4, and TPS1
Vernalization		10	ASHH3, CLF, FES1, LRB1, MAF1, MAF5, PHP, VEL1, VRN1, and WRKY34

**FIGURE 1:**
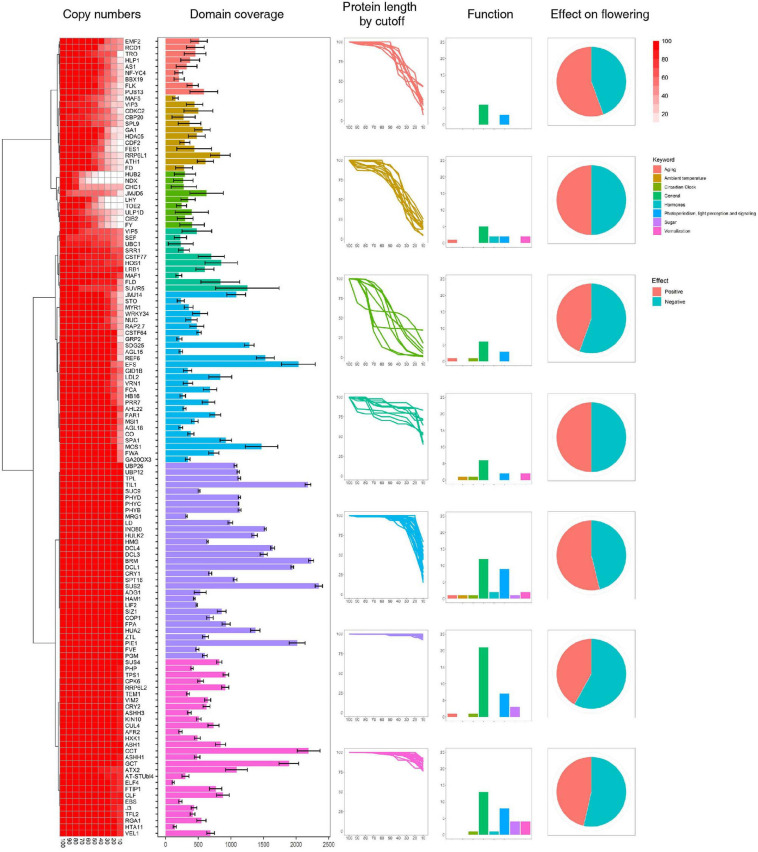
Identification of flowering-time genes. Flowering-time genes were identified by their domain architectures, and domain coverage for each gene was calculated. Functions of flowering-time genes were predicted from the FLOR-ID database.

To address this problem, we applied new method for identifying flowering-time genes using their domain architectures (Koo et al., 2020). To this end, we classified *A. thaliana* flowering-time genes into subtypes based on their domain architectures. In total, 249 flowering times genes were assigned into 131 subtypes (Supplementary Tables 2–4) and used for identification of flowering-time genes. With this approach, a total of 85,461 candidate flowering-time genes were identified from 19 species in the Malvaceae, Brassicaceae, and Solanaceae families (Figure 1). Thus, the number of flowering-time genes identified using domain architectures is increased relative to those uncovered by the BlastP method (Supplementary Table 5). In particular, genes containing a single domain with low query coverage (< 50%) were dramatically increased compared to genes containing either multiple domains or a single domain with high query coverage (Supplementary Figures 1, 2 and Supplementary Table 6). We then filtered out all genes not satisfying our criteria (i.e., protein length > 90% shorter or > 110% longer than the query gene), leaving a total of 22,784 flowering-time genes identified from 19 species. Approximately two to three percents of these genes were detected in Malvaceae genomes (Table 2).

**TABLE 2:** Flowering-time genes identified in this study.

**Family**	**Species**	**Copy numbers**	**Gene numbers of species**	**Proportion (%)**
Brassicaceae	*Arabidopsis thaliana*	757	27,655	2.74
Brassicaceae	*Brassica napus*	2,094	101,040	2.07
Brassicaceae	*Brassica oleracea*	717	35,400	2.03
Brassicaceae	*Brassica rapa*	1,071	46,221	2.32
Malvaceae	*Theobroma cacao*	537	29,452	1.82
Malvaceae	*Herrania umbratica*	521	18,848	2.76
Malvaceae	*Corchorus capsularis*	368	29,356	1.25
Malvaceae	*Durio zibethinus*	1,096	35,643	3.07
Malvaceae	*Hibiscus syriacus* (Gangneung)	2,133	87,603	2.43
Malvaceae	*Gossypium raimondii*	983	37,505	2.62
Malvaceae	*Gossypium arboreum*	968	40,960	2.36
Malvaceae	*Gossypium turneri*	795	38,871	2.05
Malvaceae	*Gossypium barbadense*	1,896	74,561	2.54
Malvaceae	*Gossypium darwinii*	1,898	78,303	2.42
Malvaceae	*Gossypium hirsutum*	1,904	75,376	2.53
Malvaceae	*Gossypium mustelinum*	1,946	74,699	2.61
Malvaceae	*Gossypium tomentosum*	1,916	78,338	2.45
Solanaceae	*Capsicum annuum* CM334	514	34,899	1.47
Solanaceae	*Solanum lycopersicum*	684	34,688	1.97
Total	-	22,798	979,418	-

To further assess the effect of domain numbers on prediction accuracy, the correlation between domain numbers and gene copy numbers was investigated (Supplementary Table 6). We found that standard deviations of domain coverage are decreased for multiple-domain genes compared to single-domain genes (Supplementary Table 6). In addition, copy numbers of common genes identified by the BlastP and domain architecture methods become similar with application of a low cutoff value. Notably, while the copy numbers of individual genes are affected by the cutoff value, the presence and absence of individual genes is not (Supplementary Table 6). Collectively, we find that multiple-domain genes show more accuracy compared to single-domain genes by both BlastP and domain architecture-based gene search methods. These results are likely due to differences in length and sequence variation that results from the increased evolutionary distance between Malvaceae and other families.

### Genes Involved in Regulation of Flowering Time or Floral Development Are More Highly Conserved Than Other Genes

The genes identified above were assigned to seven clusters based on protein length as a cutoff value (Figure 1 and Supplementary Figure 1). Genes in Clusters 6 and 7 show little length variation with changing cutoff values, whereas Cluster 1 genes show high length variation. In addition, we find that genes involved in sugar pathway and ambient temperature show little length variation (Figure 1), which might indicate high sequence conservation during evolution. To test this hypothesis, the conservation of individual genes in each cluster was assessed using conservation scores reported in a previous study (Supplementary Figure 3) (Koo et al., 2020). Our results reveal that relatively high numbers of genes involved in sugar pathway and ambient temperature remain after filtering by conservation score (> 50%) compared to genes involved in hormone signaling. Similarly, high numbers of Cluster 6 and 7 genes, which show little length variation, remain after filtering by conservation score (Supplementary Figure 4). These data suggest that length variation of orthologous genes might be correlated with their sequence conservation during evolution.

**FIGURE 2:**
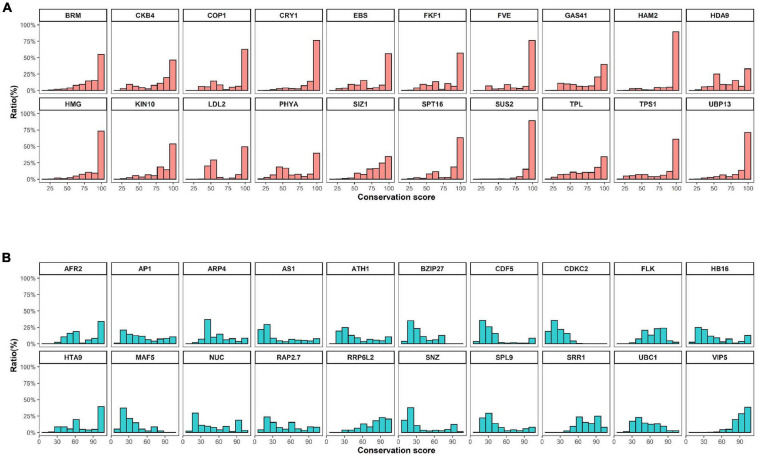
Conservation scores of conserved and variable proteins. **(A)** and **(B)** The top 20 **(A)** conserved or **(B)** variable proteins were selected by their domain architectures and filtered based on comparison of protein length with *Arabidopsis* templates. Conservation scores were calculated using methods described in a previous study (Koo et al., 2020).

High levels of presence and absence variations (PAV) were also detected in Cluster 3 at decreasing cutoff values (Figure 1). These data suggest that the high sequence variation observed in Cluster 3 genes is caused by either structural variations arising in these genes during evolution or annotation errors. To investigate the possibility of annotation errors, the conservation of core regions from representative genes showing low (phytochrome A) or high gene-length variation (*CDCK2*) were also investigated (Supplementary Figure 5). Notably, we observed high sequence conservation for core regions phytochrome A, whereas a low conservation rate was observed for core regions of CDCK2. Furthermore, little changes in conservation rates were observed at different cutoff values in phytochrome A, while increases in sequence conservation rates were observed by cutoff value in CDCK2. The core region of CDCK2 (protein kinase domain, IPR000719) shows high sequence variation rates in individual species (Figure 2), and these patterns were also observed for the conservation scores of individual CDCK2 genes with high and low length variation (Supplementary Figure 6). These data indicate that the low sequence conservation rates of genes with high levels of length variation is not caused by their length variation, but rather is due to sequence variations in their core domains. Thus, our findings suggest that the high levels of PAVs in Cluster 3 might be caused by sequence variations or structural variations of individual genomes that have led to functional diversification of core domains during evolution. Consistent with this observation, high conservation rates were also detected in Clusters 6 and 7, whereas high rates of variability were found in Clusters 1 and 2 (Supplementary Figures 7, 8).

### Recursive Polyploidization Contributed to Expansion of Flowering-Time Genes and Increases in Gene Repertoires

**FIGURE 3:**
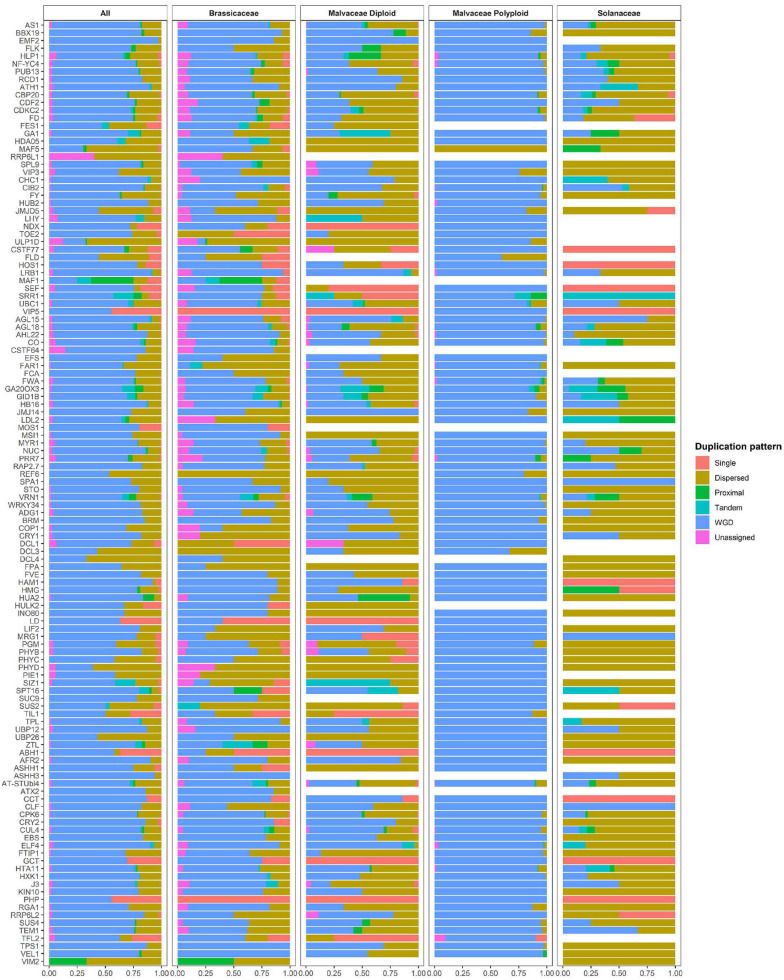
Duplication pattern analysis of flowering-time genes. Flowering-time genes were analyzed using MCScanX, and duplication patterns were classified as singleton, dispersed, proximal, tandem, segmental/whole genome duplication (WGD), or unassigned.

Polyploid genomes are common in the Malvaceae family (e.g., in *G. barbadense*, *G. darwinii*, *G. hirsutum*, *G. mustelinum*, *G. tomentosum*, and *H. syriacus*). Therefore, to investigate the relationship between polyploidization and copy numbers of flowering-time genes in Malvaceae, duplication pattern analysis was performed using MCScanX (Figure 3, Table 3 and Supplementary Table 7; Wang Y. et al., 2012). It is also known that diploidization and homolog loss occurs in polyploid genomes to maintain genome stability (Soltis et al., 2015), and gene repertoires are regulated by these polyploidization and subsequent diploidization events (Kim Y.M. et al., 2017). Therefore, we also investigated copy numbers and collinearity of flowering-time genes in polyploid Malvaceae genomes (Figure 3, Table 3 and Supplementary Table 7). Results of these analyses indicate that most duplicated flowering-time genes conserve their high copy numbers and collinearities. Among diploid genomes, polyploidization and subsequent diploidization have occurred in *G. raimondii* (Wang K. et al., 2012) and *G. arboreum* (Li et al., 2014). Here, we detected paralogous gene pairs generated following WGDs in diploid Malvaceae genomes by MCScanX analysis (Figure 3, Table 3 and Supplementary Table 7). Among the Brassicaceae genomes, which include the allotetraploid *Brassica napus*, high copy numbers of paralogous gene pairs were detected. In contrast, recent WGD events have not occurred in both Solanaceae genomes (i.e., in *S. lycopersicum* and *C. annum)*, and relatively few paralogous gene pairs were detected in this group compared to other families (Supplementary Tables 7, 8).

**TABLE 3:** Classification of duplicated genes.

**Species**	**Singleton**	**Dispersed**	**Proximal**	**Tandem**	**WGD/Segmental**	**Unassigned**	**Total**
*Arabidopsis thaliana*	78	307	20	57	295	0	757
*Brassica napus*	0	283	10	18	1,414	369	2,094
*Brassica oleracea*	8	220	15	15	459	0	717
*Brassica rapa*	11	257	13	28	761	1	1,071
*Theobroma cacao*	19	269	40	38	170	1	537
*Gossypium raimondii*	14	380	32	60	491	6	983
*Gossypium arboreum*	44	389	28	56	410	41	968
*Gossypium turneri*	17	330	24	47	377	0	795
*Gossypium barbadense*	1	100	18	16	1,732	29	1,896
*Gossypium darwinii*	0	91	20	10	1,772	5	1,898
*Gossypium hirsutum*	0	90	17	22	1,764	11	1,904
*Gossypium mustelinum*	0	89	27	21	1,804	5	1,946
*Gossypium tomentosum*	0	89	13	23	1,788	3	1,916
*Capsicum annuum* CM334	15	360	34	40	65	0	514
*Solanum lycopersicum*	18	381	41	57	187	0	684

For paralogous gene pairs, traces of diploidization evident as dispersed genes were detected for a number of genes, including MAF5, VIP3, SUC9, and FLD. In the case of FLD and SUC9, these genes were not detected in diploid parental lines but are present in tetraploid genomes. These data may indicate that loss of the FLD and SUC9 genes occurred in diploid genome of *G. arboreum* and *G. raimondii* by diploidization and homolog loss (Figure 3 and Supplementary Table 7). Conversely, HULK2 gene was only detected in the *Theobroma cacao* genome (Supplementary Table 7). These data also reveal family- or species-specific PAVs in multiple genes, such as EMF2, NDX, TOE2, ULP1D, and CSTF77. Furthermore, copy numbers and ratios of duplicated genes were found to be different in individual species and in each family (Figure 3 and Supplementary Tables 7, 8). Collectively, these data suggest that copy numbers of flowering-time genes within individual species and families were mainly increased following WGD, and subsequently, gene repertoires were determined by diploidization and natural selection to adapt to changing environmental conditions during evolution.

### Duplicated Flowering-Time Genes Were Conserved Against Genomic Structural Variations to Adapt to Environmental Changes During Evolution

Whole genome duplication plays an important role in plant survival by enhancing the potential for environmental adaptation. This adaptation might be achieved by increasing gene repertoires or functional diversification *via* neofunctionalization or subfunctionalization (Soltis et al., 2015). Therefore, we measured the ratios of duplicated genes to total genes within the groups of CRGs, drought stress-related genes, and flowering-time genes in Brassicaceae, Malvaceae, and Solanaceae (Supplementary Figure 9). We found that the ratios of flowering-time genes are similar to those of cold-related and drought stress-related genes (Supplementary Figure 9A). However, flowering-time genes display the highest ratios among all genes duplicated following WGD relative to the other two groups (Supplementary Figure 9B). These data indicate that duplicated flowering-time genes are more highly conserved relative to other gene families.

**FIGURE 4:**
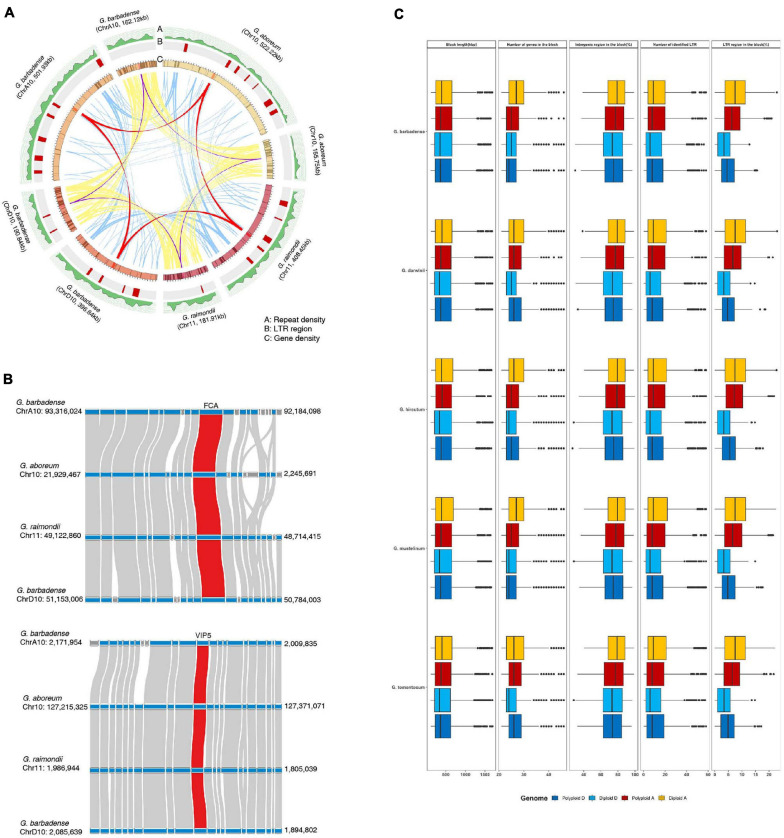
Comparison of genomic structures near flowering-time genes in diploid and polyploid genomes. **(A)** Analysis of gene collinearity and genomic alterations between A and D genomes. **(B)** Investigation of microsynteny near the FCA and VIP5 genes. **(C)** Analysis of genomic variations in flowering-time genes and their flanking regions in diploid and polyploid genomes.

However, some biologically important genes have lost their duplicated counterparts during evolution in diploid and polyploid genomes. This suggests that structural variations within the genome, including inversions, translocations, or genomic imbalances (insertions and deletions), may have played important roles in regulating copy numbers of flowering-time genes during evolution. Therefore, to test this, we measured genomic structural variations near duplicated genes, including dispersed genes that have lost their duplicated counterparts, in parental diploid genomes and polyploid genomes (Figure 4 and Supplementary Table 9). Our results indicate that most duplicated flowering-time genes are conserved, although active structural variations have occurred in their flanking regions (Figure 4). In the case of the *FCA* and *VIP5* genes, densities of repeat sequences and long terminal repeat (LTR) elements were found to differ between parental diploid and polyploid genomes (Figure 4A). These data indicate that active proliferation of LTR elements occurred after polyploidization in individual genomes. In addition, gene insertion events were detected in flanking regions of the *FCA* and *VIP5* genes (Figure 4B). Analysis of the genomic regions flanking flowering-time genes indicates that active genomic variations occurred in both diploid and polyploid genomes subsequent to WGD (Figure 4C). These data suggest that structural variations, such as active proliferation of repeat sequences and gene insertions, independently occurred in gene flanking regions, and gene deletion or alterations in expression gene patterns might have occurred in some individual plants. Furthermore, estimation of synonymous nucleotide substitutions at synonymous sites (Ks) of flowering-time genes suggested that ancient WGD before allotetraploidization occurred in *G. arboreum* and *G. raimondii* and recent WGD occurred in tetraploid genomes may be equally contributed to expansion of flowering-time genes (Supplementary Figure 10).

**FIGURE 5:**
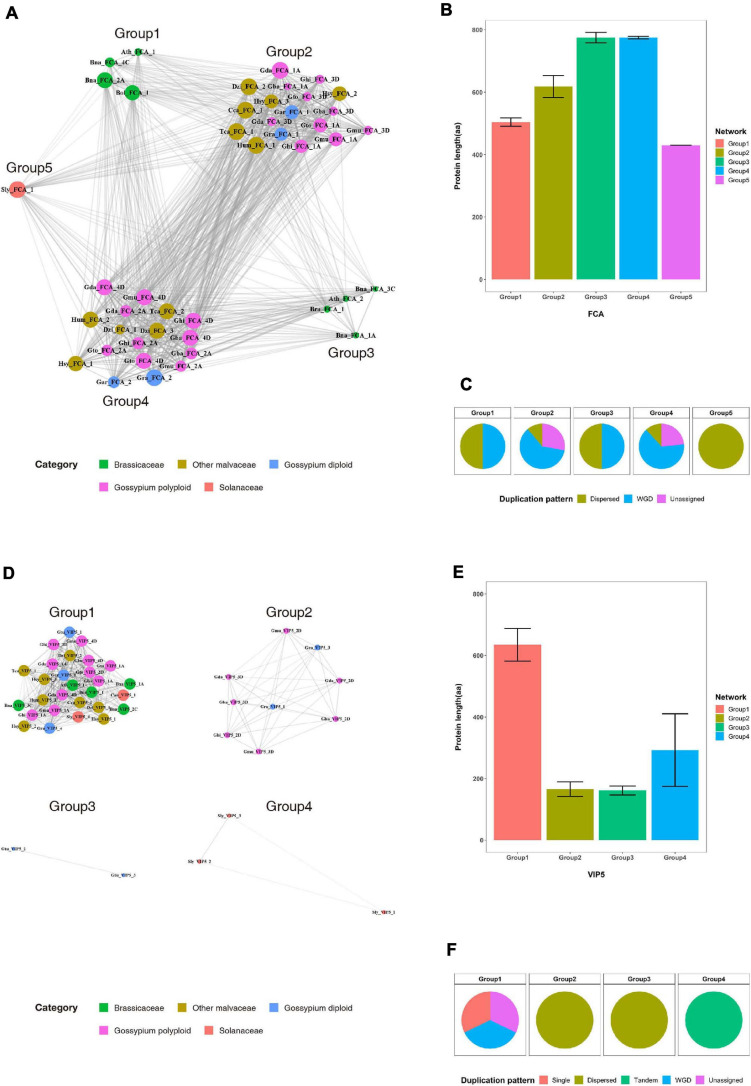
Sequence similarity network analysis for the FCA and VIP5 gene products in the Brassicaceae, Malvaceae, and Solanaceae families. **(A)** and **(D)** Sequence similarity networks of the **(A)** FCA and **(D)** VIP5 gene products. **(B)** and **(E)** Average protein lengths of **(B)** FCA and **(E)** VIP5 within individual groups. **(C)** and **(F)** Distribution of duplication patterns for **(C)** FCA and **(F)** VIP5 within individual groups.

Most duplicated flowering-time genes are conserved, suggesting they play important roles in Malvaceae species. In previous studies, functional diversification of duplicated genes *via* such processes as subfunctionalization or fractionation was detected in polyploid genomes (Dodsworth et al., 2015; Freeling et al., 2015). Here, to investigate the functional diversification of duplicated genes, sequence similarity network and phylogenetic analyses were performed with the FCA and VIP5 gene products (Figure 5 and Supplementary Figure 11). Results indicate that the FCA and VIP5 gene products can be divided into five and four major groups, respectively (Figures 5A,D), and neither the length of individual proteins (Figures 5B,D) nor duplication pattern (Figures 5C,F) affects formation of each group. Phylogenetic tree analysis further shows that the major clusters within the phylogenetic tree are consistent with clusters from our sequence similarity network analysis (Supplementary Figure 11). Collectively, our data suggest that flowering-time genes were duplicated by recursive polyploidization and have been maintained in high copy numbers during evolution to enhance adaptation against environmental stressors. These duplicated genes might subsequently have been functionally diversified within families and individual species during the course of evolution.

## Discussion

Because plants are immobile, it is vitally important for these organisms to have the ability to effectively adapt to environmental changes. Thus, polyploidization by WGD, one of the most important mechanisms for enhancing adaptation ability, is a prevalent phenomenon in plants (Wood et al., 2009). Polyploids display a selective advantage over diploids through the evolution of novel genetic variation and functions (Soltis and Soltis, 2000; Leitch and Leitch, 2008; Flagel and Wendel, 2009). Notably, recurrent processes of polyploidization have led to differential gene loss and copy number variations in related plant species (Soltis et al., 2015; Kim Y.M. et al., 2017). The flowering-time genes are one of the largest and most functionally important gene families in plants. Previous studies have shown that the largest plant gene families have been massively expanded by various genomic mechanisms, including WGD (Kim Y.M. et al., 2017; Song X.M. et al., 2020) and retroduplication (Kim S. et al., 2017). Thus, we hypothesize that flowering-time genes may have similarly been expanded following WGD and retained during evolution.

To test our hypothesis, we investigated the flowering-time genes in plants belonging to the Malvaceae, Brassicaceae, and Solanaceae families, which contain many polyploid reference genomes (Li et al., 2015; Liu et al., 2015; Yuan et al., 2015; Hu et al., 2019). For example, a number of reference genomes for polyploid plants in the genus *Gossypium* have been constructed (Li et al., 2015; Liu et al., 2015; Yuan et al., 2015; Hu et al., 2019), and the diploid genomes of *G. arboreum* and *G. raimondii* were found to have been generated following WGD, with subsequent diploidization (Wang K. et al., 2012; Li et al., 2014). Thus, genomes of the genus *Gossypium* are ideally suitable for investigating expansion of gene families following WGD. Flowering-time genes were identified in Malvaceae, Brassicaceae, and Solanaceae plants by a domain architecture-based approach using flowering-time genes in *A. thaliana* as templates (Figure 1). We note that Blast analysis was inefficient for identifying orthologous genes in plants belonging to the Malvaceae family with flowering-time genes in *A. thaliana* as queries, likely due to the large evolutionary distance between Malvaceae and Brassicaceae.

To improve accuracy of identification, we filtered our results based on protein length of candidate genes, resulting in the identification of 22,784 flowering-time genes from 19 species. Genes were divided into seven clusters based on patterns of protein length variations, and these were correlated with conservation of sequences (Figure 1 and Supplementary Figures 7, 8). In addition, we found that low conservation scores for core domains of genes with high length variation suggests accumulation of genomic variations and functional diversifications of these genes during evolution (Supplementary Figure 6). MCScanX analysis was then performed to investigate gene duplication mechanisms (Figure 3). Our results indicate that flowering-time genes were mainly duplicated following WGD, and most paralogs of flowering-time genes are detected in polyploid genomes (Figure 3). In addition, many paralogous genes for those duplicated following WGD can be detected in diploid genomes in Malvaceae and Brassicaceae. As diploid genomes in the genus *Gossypium*, *Brassica rapa* (Wang X. et al., 2011) were also diploid genomes occurred in WGD.

In general, the legacy of polyploidization is masked by subsequent diploidization, including large-scale genome reorganizations involving repetitive DNA loss, chromosome rearrangements (e.g., fusions and fissions), and complex patterns of gene loss (Dodsworth et al., 2015; Soltis et al., 2015). Furthermore, differential retention of duplicated genes during the process of diploidization is observed across different gene classes, with some duplicated genes more prone to retention than others (Dodsworth et al., 2015; Soltis et al., 2015). In previous studies assessing survival rates of duplicated genes, functionally important genes have been shown to be more prone to retention. To test this here, the flanking regions of flowering-time genes were investigated in polyploid genomes and in two parental diploid genomes. Our results indicate that most duplicated flowering-time genes have been retained, although proliferation of repetitive sequences and gene insertions are detected in their flanking regions (Figure 4 and Supplementary Table 9). The estimation of Ks value of flowering-time genes also suggested that duplicated genes following two WGDs were retained in diploid and polyploid genomes (Supplementary Figure 10). Thus, these data suggest that flowering-time genes play important roles in adaptation to environmental changes, and consequently, these genes have been retained during evolution. Previous studies have shown that the multiple copies of genes duplicated following WGD are functionally diversified by neofunctionalization or subfunctionalization (Dodsworth et al., 2015; Soltis et al., 2015). In addition, approximately 50% of paralogs following WGD had undergone subfunctionalization (Roulin et al., 2013). Here, sequence similarity network and phylogenetic tree analyses indicate that duplicated flowering-time genes form a number of major clusters, thus suggesting the functional diversification of these duplicated genes (Figure 5 and Supplementary Figure 11). As functionally diversified genes play important roles in plant development and provide advantages to plants for adaptation or survival against environmental changes during evolution, the flowering-time genes have been retained as large and diverse gene family.

Overall, our findings in this study suggest that the flowering-time genes were mainly expanded following WGD and subsequently retained during evolution due to their functional importance for survival in plants. In addition, the multiple copies of duplicated genes were functionally diversified and have contributed to the formation of distinctive phenotypes for individual species.

## Data Availability Statement

The datasets presented in this study can be found in online repositories. The names of the repository/repositories and accession number(s) can be found in the article/Supplementary Material.

## Author Contributions

A-YS and Y-MK wrote the manuscript, conceived the project, designed the experiments, and organized the manuscript. SH, YL, S-YK, and A-YS generated the data and performed bioinformatic analyses. All authors contributed to the article and approved the submitted version.

## Conflict of Interest

The authors declare that the research was conducted in the absence of any commercial or financial relationships that could be construed as a potential conflict of interest.

## Footnotes

1 www.ncbi.nlm.nih.gov/assembly/GCF_002168275.1

## Supplementary Material

The Supplementary Material for this article can be found on Figshare. Supplementary Tables 1 and 5, and Supplementary Figures 1–11 (doi: 10.6084/m9.figshare.14822070). Supplementary Tables 2–4 and 6–9 (doi: 10.6084/m9.figshare.14822061).
